# Memoirs of the Life and Medical Opinions of Dr. Armstrong

**Published:** 1835-01-01

**Authors:** 


### Memoir of the Life and Medical Opinions of John Armstrong, M.D. to which is added an Enquiry into the Facts connected with those Forms of Fever attributed to Malaria or Marsh Effluvium. By *Francis Boott*, M.D. Secretary to the Linnæan Society, &c. &c. Second Volume, 8vo. pp. 752, 1834.

In a former number of this Journal we gave some account of the first volume of Dr. Boott's work, more especially of that part of it which related to the life and death of our old and esteemed friend Dr. John Armstrong. We did not enter on the wide range of enquiry respecting the nature and causes of fever, commenced in the first volume and concluded in this the second; deterred, partly by the difficulty, partly by the extent of the investigation. Even now, our moral courage is not "screwed up to the sticking point;" and while we admire the talent, we still more admire the patience with which Dr. Boott has pursued a subject that may be said to be at once exhausted and untouched. The labourers in this fertile field of inquiry have reaped little more than if they had been tilling the sands of Africa, or ploughing the wavy ocean. Their tracks have been obliterated, one after the other, as the footsteps of the traveller are effaced by the first gale in the desert, or the wake of the scudding bark by the rolling waves that pursue her.

In the course of enquiry into the causes of fever, by various authors, during the last fifty years, one thing is clear, that more and more stress is daily laid on terrestro-aerial causes, and less on specific contagions. While some inquirers (for instance, Dr. Maclean and others) have denied that plague itself originates from contact or contagion, the greater part of the more close observers have limited very much the range of this dreaded agent, and viewed fevers, as well as many other diseases, as generally of an epidemic nature, and only occasionally taking on the adventitious power of propagating themselves from person to person, independent of the primary or essential cause. Our present author appears to go beyond this median line, and to incline much towards the ultra tenets of the Maclean school. We acknowledge, too, that he supports his tenets with no less ability than ingenuity. He commences his investigation with the plague of the Levant, and thinks he can shew that—"it is a malarious disease, depending on those inscrutable causes which give an epidemic prevalence and intensity to specific fever." He remarks that, if many men of great talent and observation contend that plague is propagated, like small-pox, from person to person, the same was long contended for in the case of the yellow fever of America, which is now almost universally allowed to be an endemic disease, dependent on locality and temperature. Sydenham and Armstrong are referred to, as favouring the idea that plague bears a close analogy to typhus.

"The views of Dr. Armstrong with respect to the general character of marsh fever, if applied to plague, would remove much of the uncertainty which has prevailed as to the pathological conditions of its various forms, and would lead to the adoption of a more rational treatment; and to shew that these views were applicable to it, was the motive which induced him to make it the subject of a separate essay. He was of opinion that it was a very aggravated form of typhus, arising, like it, originally from malaria, characterised by the same general conditions, and to be treated on the same principles; that its intensity depended upon those occasional differences in the concentration of the remote cause, and upon that variable state of predisposition which is connected with seasons, habits and the changeable circumstances of life; that while, in every epidemic of plague, cases were observed not distinguishable from the typhus of this country, so here, in severe visitations of the epidemic prevalence of the last, instances were occasionally met with which approached in character to the true plague. In those countries where plague is frequently epidemic, it is not surprising, judging from the analogy of yellow fever, that it should assume a variable character. In this country, typhus has a narrower range, because there are no circumstances favouring its most aggravated forms, so that it is only at long intervals, and in individual cases, that it assumes a malignancy like that of plague. In the South of Europe, Asia and Africa, these circumstances more commonly occur, and every gradation of effect is met with. 'The plague,' says Sir A. B. Faulkner, 'above every other distemper with which I am acquainted, either by reading or experience, is one of the most irregular type, modified in its symptoms and appearances to a degree surpassing all belief, and every attempt to explain by difference of constitution, age, temperament, manner of life, and other peculiarities in its victims. The character of the concomitant fever becomes extremely irregular, assuming every shade of variety, from synocha down to the lowest degree of typhus, and in some instances having accessions of rigor not unlike an irregular species of intermittent.'" 6.

Our author conceives that "the doctrine of contagion fettered the mind of Bancroft, and prevented his taking those enlarged views with regard to typhus and plague, which he has forcibly applied to yellow fever."

"The existence of an intermitting and remitting type of fever in plague, which was observed by Price and Sir James M'Gregor in Egypt, is attempted to be explained by Bancroft on the supposition of the joint action of malaria and the contagion of plague, just as Chisholm accounted for his Bulam fever by associating malaria with the contagion of typhus. This idea of two distinct causes co-operating to produce a compound disease arose among the advocates of contagion, who were otherwise unable to explain the occurrence of intermitting fever in epidemics, which ultimately assume a continued typhoid character. Pringle endeavoured on this supposition to explain the Hungarian fever of the sixteenth century, from the belief that marsh exhalations alone could give rise to the periodical types of fever, and that the malignant continued form was the exclusive progeny of human effluvia." 7.

Dr. Boott, as an attentive observer of facts, must be aware that *almost* every disease is influenced and modified by the epidemic constitution of the day, as well as by locality and climate. Why then should not plague take on a remittent, or even intermittent form occasionally, when malaria, the grand source of intermittent affections, prevails in an unusual degree? The following argument is, in our opinion, more germane to the subject.

"The affection of the glandular system, though considered as pathognomonic of plague, is not universal in that disease, nor confined exclusively to it. We have occasional evidence, not only of buboes and carbuncles, but of exanthemata in typhus and yellow fever; and, however difficult it may be to account for the comparative infrequency of the two former affections in the latter diseases, their occasional occurrence is a subject of important consideration, as adding some weight to the idea of the three diseases being mutually allied. It is only in the worst cases of typhus that these more frequent symptoms of plague make their appearance, which would lead to the inference, that the one disease is a slighter modification of the other; an impression strengthened by the character of the fever recorded by Sydenham as having preceded and followed the plague of London in 1665. It is impossible to account for the disappearance of plague in this country on the idea that it was formerly imported into it, considering how frequently those morbid states of atmosphere must have occurred within the last hundred and fifty years to favour the reception and diffusion of the contagious principle, and the greater intercourse which the multiplied commercial dealings of modern times have established with the Levant; and though it may be equally difficult to explain why endemic causes, if they ever gave origin to plague in England, have not produced it since 1679, yet some portion of that difficulty is removed, on the supposition that typhus is the same disease modified by circumstances favouring the general condition of the people, and only appearing occasionally in severe epidemics with those symptoms which more particularly belong to plague." 9.

The whole of a large volume is dedicated to the illustration of this alliance between plague, yellow fever, and typhus; and, in thirteen chapters the subject is discussed with great acumen, patience, judgment, and research. This discussion is in itself an extended review and analysis of various writings, ancient and modern, which it would be utterly impossible to review or embrace in an article less extended than a whole number of this Journal. We must therefore pass over twelve out of the thirteen chapters of the work, in order to dedicate some space and attention to the concluding one—the Fevers of Great Britain.

In this section the author refers chiefly to the writings of Willan and Bateman for evidence of the character of typhus in London, contrasting their observations with those of Huxham—and finally appealing to the able reports of the Irish physicians during the epidemic of 1817, to shew that the origin of that scourge was almost universally spontaneous, while its mortality was trifling compared with the epidemics of Paris, which occurred between the years 1822 and 1827, and which have been so accurately described by M. Louis, in two volumes, 8vo. 1829. Willan has furnished some data shewing the proportion, or rather disproportion, between continued and periodical fevers in London, from 1796 to 1800 inclusive. The general result is as follows ;—

| | Intermittents. | Summer bilious. | Slow. | Malignant. |
|---|---|---|---|---|
| 1796 (9 months.) | 26 | 22 | 14 | 32 |
| 1797 .......... | 13 | 20 | 21 | 51 |
| 1798 .......... | 9 | 24 | 26 | 74 |
| 1799 .......... | 8 | 28 | 32 | 99 |
| 1800 .......... | 12 | 38 | 43 | 316 |

By the above table it appears that, in 1796, the intermittents were to the slow and malignant as 26 to 46 ;—while in the year 1800, they were as 12 to 359, or about 1 to 30! Dr. W. appears to consider the intermittents as essentially distinct from the continued malignant fevers, but, as Dr. Boott justly observes,

"When we find that the one prevails to the exclusion of the other in different countries, each in some respects obedient to seasons, essentially perhaps autumnal diseases, as far as their maximum prevalence is concerned, heat giving an earlier development to the one, and cold protracting the duration of the other into winter, though often as effectually checked by frost; and especially when we observe the transition of the one type into the other, we are led to believe that they are but modifications of one disease." 593.

Dr. Fothergill, indeed, in 1753, speaks of spring-fevers which, by moderate evacuations, became regular intermittents, and were cured by bark. The same he observes of summer remittents, after bleeding and emetics, becoming intermittents. "It is transitions of this nature," says Dr. B. "that justify the idea of the types of fever being modifications of one disease, varying in the same individual, and frequently assuming a difference of character in successive seasons, gradually approximating to a typhoid form, without any difference of cause beyond atmospheric influences." In 1796, between the 1st of January and the 20th of March, Willan reports the existence of much "*slow fever*," which did not arise from contagion, and did not take on any contagious character during its course. Our author takes a wide review of the fevers of this country, and then proceeds to a similar investigation of the epidemics in Ireland. We cannot follow him, but we strongly recommend the work to the candid perusal of our brethren. With the following extract we must conclude.

"That typhus universally and exclusively depends on contagion for its origin and diffusion, the testimony of the Irish physicians, in Table No. 5, sufficiently disproves; and as Bateman and many other English authorities admit of its spontaneous origin, the question as to this point must be considered as set at rest. Whether, so arising, it is ever propagated by a contagion *sui generis*, is a circumstance that future inquiry must determine; for though the balance of opinion is very general in its favour, the mind of the profession, so recently and so universally absorbed by the doctrine of exclusive contagion, is not unbiassed enough at present to decide upon it. That great changes have occurred in medical opinion on the subject of fever within the last 15 or 20 years, is apparent from the doctrines of the exclusive contagiousness and debility of typhus having been very generally abandoned, and from the change that has taken place with regard to the causes of yellow fever; and it cannot be doubted that the spirit of free enquiry which is abroad will lead to essential modifications in the opinion that now prevails as to the causes which contribute to the diffusion of typhus." 682.

The design of Dr. Boott is clearly to lessen the extent of our belief in the doctrine of contagion, as the cause of fever, and to direct our attention more to its terrestro-aërial etiology. We are of opinion that a candid and careful perusal of these volumes will tend very much to accomplish the object he had in their construction. At all events, the author has proved himself to be a man of extended views, of unprejudiced mind, and of ample information. The profession is under much obligation to him for the pains which he has taken to compare and compile such a great mass of materials.

